# Closing the Gap: Horizontal Transfer of *Mariner* Transposons between *Rhus* Gall Aphids and Other Insects

**DOI:** 10.3390/biology11050731

**Published:** 2022-05-10

**Authors:** Aftab Ahmad, Xu Su, AJ Harris, Zhumei Ren

**Affiliations:** 1 School of Life Science, Shanxi University, Taiyuan 030006, China; aftabahamadkhan003@gmail.com; 2 Academy of Plateau Science and Sustainability, Qinghai Normal University, Xining 810016, China; xusu8527972@126.com; 3 School of Life Sciences, Qinghai Normal University, Xining 810008, China; 4 South China Botanical Garden, Chinese Academy of Sciences, Tianhe District, Guangzhou 510650, China; aj.harris@inbox.com

**Keywords:** horizontal transfer, *Rhus* gall aphids, *Mariner* transposable elements

## Abstract

**Simple Summary:**

Transposable elements (TEs) are mobile genetic elements that invade and multiply in host genomes. Besides vertical inheritance, it can transfer from one species to another through a phenomenon called horizontal transfer (HT). HT is crucial for TEs survival in genomes but also a significant disadvantage for host genomes, and recurrent HTT events between different hosts could shape and affect their genome architecture. HTT could be harmful to host genomes, but sometimes it can be useful and may play a role in the adaptive evolution of the host genome. HTT is well reported in many eukaryotes; however, there is still a considerable gap of knowledge about HTT in some organisms. This study closed the knowledge gap about HTT regarding *Rhus* gall aphids and reported multiple events of HTT involving *Rhus* gall aphids and other insects.

**Abstract:**

Horizontal transfer of transposons (HTT) is an essential source of genomic evolution in eukaryotes. The HTT dynamics are well characterized in eukaryotes, including insects; however, there is a considerable gap in knowledge about HTT regarding many eukaryotes’ species. In this study, we analyzed the events of the HTT between *Rhus* gall aphids (Hemiptera) and other insects. We analyzed the *Mariner*-like transposable elements (MLEs) belonging to *Rhus* gall aphids for the possible HT events. The MLEs have a patchy distribution and high similarity over the entire element length with insect MLEs from different orders. We selected representative sequences from the *Rhus* gall MLEs and identified five events of HT between MLEs of *Rhus* gall aphids and other insects from five different orders. We also found multiple HTT events among the MLEs of insects from the five orders, demonstrating that these *Mariner* elements have been involved in recurrent HT between *Rhus* gall aphids and other insects. Our current study closed the knowledge gap surrounding HTT and reported the events between *Rhus* gall aphids and other insects for the first time. We believe that this study about HTT events will help us understand the evolution and spread of transposable elements in the genomes of *Rhus* gall aphids.

## 1. Introduction

Transposable elements (TEs) are mobile DNA sequences that can translocate in the host genome and replicate their number. This ability of TEs allows them to invade virtually all kinds of organisms, from prokaryotes to higher vertebrates, and including those of humans [1] and plants [2,3]. However, the mobility of TEs can lead to significant adaptive changes by promoting chromosomal rearrangements such as segmental duplications, deletions, and inversions through phenomena such as non-allelic homologous recombination [4,5]. On the other hand, TEs’ expansion in the host genomes can also be harmful, leading eukaryotes to evolve various defense and regulatory mechanisms [6]. Due to their mobile self-regulatory activity, TEs have many biotechnological implications. They can be used as an expression vector and a vector in gene therapy, e.g., sleeping beauty transposon system [7,8].

Like all other nuclear genes, TEs can also be inherited vertically, from parents to offspring; however, TEs can be transmitted among different organisms through a phenomenon known as horizontal transfer (HT). HT can have an immediate or delayed effect on the host organism [9]. The exact mechanisms and pathway of HT of TEs (HTT) are not well understood, though it is most certainly related to the mobile nature of TEs, as the normal genes, in comparison, are much more rarely found to be transferred horizontally in eukaryotes [10,11,12,13,14]. HTT is typically inferred when the nucleotide divergence between TE copies from two distantly related hosts is much lower than expected due to vertical inheritance since the last common ancestor of the two hosts [15].

TEs are classified into two classes based on their transposition mechanism: Class I elements, or retrotransposons, move by a copy–paste mechanism and Class II elements, or DNA transposons, move through a DNA intermediate [16]. HT appears to be more persistent in DNA transposons. The *Tc1/Mariner* superfamily is the most common type to be transferred horizontally, and many studies showed the prevalence of *Tc1/Mariner* TEs in HTT among diverse animal taxon [17,18,19,20]. The underlying mechanism and vectors involved in HTTs are unclear; however, recent studies have speculated hypotheses and indirect evidence of HTT species [21]. With the advancement and invention of new technologies, and the significant scale analysis of different organisms genome shows that several host and parasitic features can be considered to facilitate the occurrence of HTT, including the occurrence of some parasites with multiple host species. At the same time, a symbiotic association between different species could also leverage the phenomena of HTT among some species [21]. The TE research community has evaluated many vectors with little or no success, while some studies have hypothesized that parasites can mediate the transfer of TEs from one species to another. Some recent studies have proposed that viruses can be the possible vectors that mediate HTT [22,23,24,25].

Class Insecta has one of the most extensive species diversity on earth and represents one of the main eukaryotic evolutionary branches. Insect genomes have been studied extensively to detect TEs, and several HTTs events have been reported in insects [26,27,28,29,30], including the first case of HTTs of P elements in *Drosophila* [15]. A recent study reported more than two thousand HTT events among 195 insect species and closed significant gaps related to insects’ HTT occurrence [19]. However, no HTT events related to gall-forming aphids were reported due to the unavailability of their genomes and TEs in the public database. Although HTT is well studied in most insects and some aphid species [31,32,33], there is no information about HTT events in the genome of galling aphids. We recently uncovered the existence of *Mariner* transposons in seven species of *Rhus* gall aphids [34], while phylogenetic analysis of detected TEs showed patchy distribution, predicting the occurrence of HT among *Rhus* gall aphids and organisms belonging to different insect orders. We performed a detailed analysis of *Mariner*-like transposable elements (MLEs) in *Rhus* gall aphids in the present study. We unveiled for the first time several events of HTT between the *Rhus* gall aphid genome and other insects.

*Rhus* gall aphids (Aphidoidea: Eriosomatinae: Fordini) are sap-feeding aphids and parasitized plant hosts of the *Rhus* genus. In contrast to other aphids, *Rhus* gall aphids are not very harmful and do not damage the host plant. Recent studies have reported a symbiotic association and complex nutrient exchange between these gall-forming aphids and plants [35]. *Rhus* gall aphids need to alternate between two hosts to finish their life cycle, i.e., the *Rhus* plants as the primary hosts and a few moss species as secondary hosts. They induce gall in their primary host plant, *Rhus* species (Anacardiaceae), and live inside the gall for several generations. The galls formed by these aphids are rich in tannin, which can be used in medicines, tanning, and military industry; hence, they have practical economic importance [35,36,37].

In our previous study, we reported the diversity of MLEs in seven species of *Rhus* gall aphids, i.e., *Schlechtendalia chinensis*, *Schlechtendalia peitans*, *Nurudea ibofushi*, *Melaphis rhois*, *Meitanaphis flavogallis*, *Floraphis choui*, and *Kaburagia rhusicola*. The seven species of aphids belonged to six major genera of *Rhus* gall aphids [36]. The *Mariner* family of DNA transposons is well known to perform recurrent and successful horizontal transfers, as supported by previous studies’ conclusions [17,38]. The present study aimed to analyze all MLEs detected in *Rhus* gall aphids for possible HTTs between the *Rhus* gall aphids and other insects. This study will present the first report of HTTs between *Rhus* gall aphids and other insects belonging to different orders of Class Insecta.

## 2. Materials and Methods

### 2.1. Data Availability

We used 121 *Mariner* transposons sequences as queries in databases, and the detailed information, including accession numbers, is shown in Supplementary File S1. The qualified MLEs for the HT analysis in this study constitute the set detected in seven *Rhus* gall aphid species and are available on GenBank as of August 2021. We built a species phylogenetic tree based on 20 genes downloaded from GenBank (Supplementary File S2), while *Mariner*-like elements for the target insect’s species were extracted from genome assemblies at NCBI after Blastn search and from TEs databases (Supplementary File S3). Alignment files used to obtain all the trees reported in this study can also be found in Supplementary File S4.

### 2.2. Identification and Annotation of MLEs in Targeted Species

To infer any possible transfer of *Rhus* gall MLEs with other insects, we followed the widely used two-step approach in reference to the previous studies [38]. Homology-based strategies were followed to find and extract similar nucleotide sequences from the genome of the target species. For this purpose, we used the transposable elements database, i.e., RepBase [39], and the whole genomes database, i.e., NCBI GenBank. RepBase searches for homologous sequences were performed using the “CENSOR” tool [40], implemented in RepBase [39] using the default parameters against the whole database. We retrieved a few sequences from the Repbase database and extracted sequences that followed our designed criteria (Sequence similarity ≥ 85%, and Query coverage ≥ 80%). At the same time, Blastn searches at NCBI were conducted with default parameters using MLEs of *Rhus* gall aphids in queries. We extracted the resulting Blastn hit produced by the qualified MLEs from the genome of the insect’s species and were manually analyzed for their terminal inverted repeats (TIRs). To confirm the placement of the extracted sequences in the *Mariner* family, we searched the conserved motifs and domain of the sequences, i.e., Helix-turn-Helix HTH DNA binding motif and DDE catalytic domain using CD-search [41,42] and motif search online, last accessed on September 15, 2021. The open reading frame (ORF) was also predicted using the ORF finder (implemented in Geneious prime), and the MLEs were annotated using Geneious prime v11.1 [43].

The nucleotide sequence’s identity between the MLEs of *Rhus* gall aphids and other insects included in the present study was searched for using Blastn. We used MLEs sequences with a query coverage ≥ 80%, and sequences shorter than this were filtered out from the analysis to avoid false-positive results.

### 2.3. Phylogenetic Analysis

We achieved the phylogenetic analysis in four steps: first, we aligned the full-length nucleotide sequences of all of the 121 MLEs of *Rhus* gall aphids with MAFFT v.7.1.1 [44] with the default parameter. The alignment was manually curated, followed by the construction of the ML phylogenetic tree. We used jModelTest v2.1.10 [45,46] to select the best evolutionary model that fitted adequately and resulted in a good tree. The *Rhus* gall MLEs were already classified into four subfamilies and sub-lineages in our previous study [34], and the same classification was followed in the present study.

Second, we aligned the qualified sequences from *Rhus* gall aphids and the extracted sequences from the targeted species, i.e., other insect orders using MAFFT implemented in Geneious. Alignment was trimmed manually, and MLEs phylogenetic tree was constructed with IQTREE using model GTR+I+G, as suggested by jModelTest.

In the third step, we constructed the species phylogenetic tree of seven species of *Rhus* gall aphid and the targeted insects belonging to four different orders, using fifteen mitochondrial genes and five nuclear genes. We aligned all of the 20 genes with MAFFT implemented in Geneious with default parameters. The aligned sequences were manually curated, and ML phylogenetic tree was constructed with IQTREE using model GTR+I+G, suggested by jModelTest. The tree was visualized and modified using Figtree v1.3.1 software [47], and bootstrap (1000 replicates) was used as statistical support for each branch. All the trees constructed were unrooted, which were manually rooted at the midpoint, during visualization and modification using Figtree v1.3.1.

In the fourth step, we estimated the divergence time between the species to infer the HT between the species, especially the five different clades representing each order. Divergence time was estimated using TimeTree online.

### 2.4. Estimating the Minimal Number of Horizontal Transfer Events

To infer multiple events of HTs, we analyzed and estimated the minimum number of HTT events in our present study. We considered the possibility that a single HT event may be sufficient to explain several cases of shared MLEs through horizontal transfer if they happened in the common ancestor of recently diverged species. We evaluated and compared the species tree and MLEs tree, considering all of the nodes in the tree, and predicted one HTT event if the descending clades sharing the same MLE were connected by a common ancestor.

Strictly speaking, we inferred and concluded that most of the HT events took place in ancestor branches, which passed to the descendant through a vertical transfer with slight divergence under natural selection. Nevertheless, for simplicity, we approximated that the species of our sample (*Rhus* gall aphids), whose MLEs have the closest similarity with MLEs of the other species, were potentially involved in HTT. We estimated the confirmed minimal HTT events between *Rhus* gall aphids and other insects following this procedure.

The MLEs involved in the event of HT were present in the middle of genomic contigs and chromosomes of the studied species. Their flanking sequences at both ends have minimal similarity, which rules out any possible contamination and validates the event of HT.

## 3. Results

In this study, we analyzed the MLEs that we detected in our previous study for HTT events between *Rhus* gall aphids and other insects belonging to different orders. To further understand the origin of evolution and inheritance of MLEs in *Rhus* gall aphids, we performed a detailed comparative phylogenetic analysis of the *Rhus* gall aphids and other insects’ MLEs. Many MLEs of *Rhus* gall aphids from different lineages showed high pairwise identity with the TEs of phylogenetically distantly related insect species. The unique identities between TEs of *Rhus* gall aphids and other insects prompted us to have a systematic search for the HTT events involving *Rhus* gall aphids *Mariner* transposons. Many studies have successfully documented thousands of HTT events among the species of Class Insecta [19,48]. Nevertheless, due to the lack of TEs data about *Rhus* gall aphids, there was no evidence of HTT between *Rhus* gall aphids and other insects. We followed a detailed two-step approach to discriminate the HTT event in the present studies based on homology or nucleotide sequence identity and species phylogenetic tree comparisons with MLEs phylogenetic tree. We found several events of HTT between *Rhus* gall aphids and species from five different orders of Class Insecta.

### 3.1. Phylogenetic Relationship of the Rhus Gall Aphids MLEs

**Figure 1:**
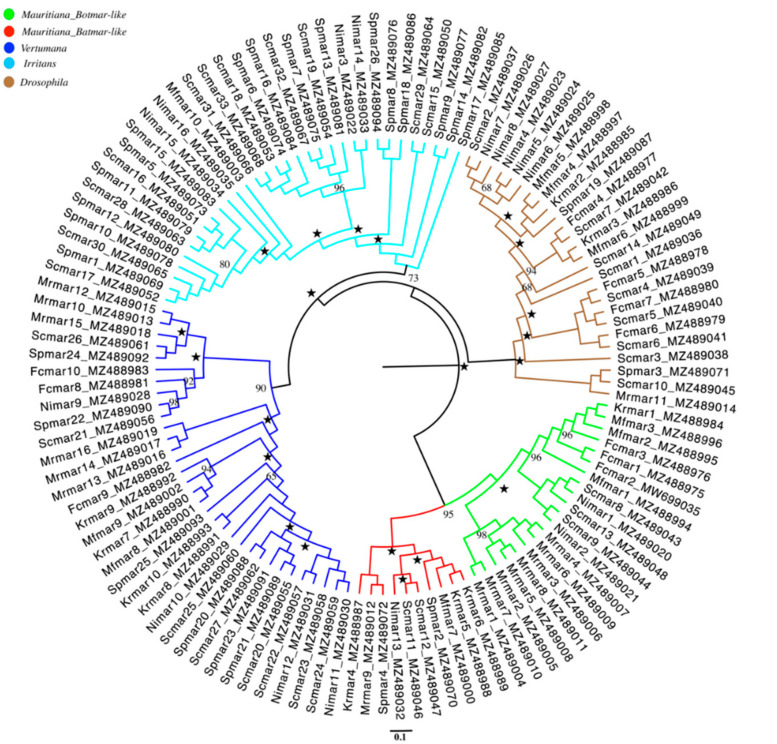
Phylogenetic tree and lineage information of all MLEs of *Rhus* gall aphids for HTT analysis. Tips of branches indicate MLE name followed by GenBank accession number. Five-pointed star represents 100% bootstrap value. The tip of branches is labeled with the first two letters representing the acronym of their scientific names, while the next three-letter followed by digits indicates the *Mariner* family name and no. of MLEs.

We determined the phylogenetic relationship of all of the MLEs of *Rhus* gall aphids analyzed in the present study with already known MLEs from the *Tcl/Mariner* Superfamily in our previous study [34]. All of the MLEs in this study belonged to four subfamilies of the *Mariner* family, i.e., *Mauritiana*, *Irritans*, *Vertumana*, and *Drosophila*. Phylogenetically, we classified MLEs of the *Mauritiana* subfamily into two sub-lineages based on sequence similarities, i.e., *Botmar-like* elements reported in *Bombyx mori* for the first time and *Batmar-like* elements found in *Bactrocera tryoni*. Phylogenetic distribution of all MLEs used in this study can be seen in Figure 1 across the seven species of *Rhus* gall aphids, among which *Mauritiana*, *Vertumana*, and *Drosophila* subfamily are distributed in all of the seven species. In comparison, MLEs from the *Irritans* subfamily is present in four species, i.e., *Schlechtendalia chinensis*, *Schlechtendalia peitans*, *Nurudea ibofushi,* and *Meitanaphis flavogallis*.

In conclusion, the phylogenetic analysis shows the distribution of DNA transposons of the *Mariner* family in all of the studied *Rhus* gall aphids species used for HTT analysis in the present study. All of the MLEs reported in seven species of *Rhus* gall aphids showed patchy distribution and are not congruent with the species phylogenetic tree, i.e., MLEs from *Irritans* are present in only four species, which might also reflect HTT events within *Rhus* gall aphid species. To infer the horizontal transfer events, we selected representative sequences from each lineage of *Rhus* gall aphids and searched for identical sequences in other insect species following homology-based approaches.

### 3.2. Selection of the MLEs Representative Sequence for HTT

Initially, to infer the HTT event between transposons of *Rhus* gall aphids and other species, specifically insects, we include all of the 121 *Rhus* gall MLEs as queries (Supplementary File S1). We blast the MLEs against the NCBI standard database and extensively search the highly similar DNA sequences in distantly related genomes. Among 121 sequences, 40 produced an excellent hit against genomes of other species with query coverage > 90% and similarity ≥ 80%. Although it is possible to include all copies of MLEs detected within one species, most sequences from the same lineages result in similar hits. Hence, it is simpler and quicker to use only a few representatives. As one sequence from the same lineage (subfamily) is enough to infer the desired result, we chose one representative from each lineage or more than one where required in each of the seven species. We selected the complete sequence from each lineage from all of the seven species, which could result in good Blastn hits (Query >90%, similarity ≥80%), and truncated sequences were discarded from the analysis. To further simplify our search and choose the best representative among the complete copies from the same lineages in each species, we chose the sequence which resulted in the best Blastn hit (high similarity and coverage with query sequence). For instance, Fcmar1, Fcmar2, and Fcmar3 belong to the same lineage of MLEs in *Floraphis choui* and resulted in similar hits, so we discarded Fcmar3 as it was incomplete, and Fcmar2 was selected as its results best Blastn hit against the genome (NCBI standard database). The same rule was applied to MLEs of all seven species, and, finally, 16 MLEs (Fcmar2, Krmar1, Krmar4, Krmar5, Mfmar1, Mfmar7, Mrmar9, Mrmar11, Mrmar16, Nimar1, Nimar13, Scmar2, Scmar10, Spmar2, Spmar3, and Spmar4) were selected.

Furthermore, to avoid repetitions and false-positive HTT events among *Rhus* gall aphids and other non-related species, only one orthologous sequence (MLE) was selected as representative from all of the MLEs in seven different species following the rules explained above. For instance, Fcmar2, Krmar1, Mfmar1, Nimar1, and Scmar2 belong to the same lineage (Figure 1), i.e., the *Botmar-like* elements of *Mauritiana* subfamily result in similar hits in Blastn, so only one Fcmar2 with the best hit was selected as representative for this lineage. The same rule was applied to all four subfamilies; for example, the three elements from the *Drosophila* subfamily, i.e., Mrmar11, Scmar10, and Spmar3, produced similar hits, so only one Mrmar11 was selected as representative for this lineage.

In some cases, more than one representative sequence was selected from the same lineage based on the Blastn hit it produced. For instance, Krmar4 and Krmar5 are derived from *Kaburagia rhusicola* and belongs to Batmar-like lineage of *Mauritiana* subfamily, but produced different Blastn hits (see Table 1), so both are included in the analysis. Finally, we selected eight MLEs from four different subfamilies involved in the HTT event between *Rhus* gall aphids and other insects. We followed the above-discussed criteria again to search for similar MLEs sequences in RepBase, which could be involved in HTT with *Rhus* gall aphids.

### 3.3. Inference of Horizontal Transfer between Rhus Gall TEs with Other Insects

Horizontal transfer event is well documented in many species of insects, but there are no reports of HTT of transposons in *Rhus* gall aphids. HT of transposons can be conferred either based on DNA sequence similarities or phylogenetic incongruences of TEs compared to neutrally evolving vertically transmitted genes or by combining both methods. Sequences of distantly related species with query coverage and similarity > 90% are considered to be horizontally transferred [48], while sequences sharing terminal inverted repeats (TIRs) of a similarity > 90% could also be a result of HTT event [16]. We also followed the commonly used two-step approach to unveil the phenomena of HT in *Rhus* gall aphids and other insects.

### 3.4. Inference of HTT Based on Nucleotides Sequence Similarities

The first step to uncovering the possible events of HT in *Rhus* gall aphids was based on DNA sequence similarities with other non-related species. We search for homologous sequences of the *Rhus* gall MLEs in Repbase using the “censor” tool implemented in the Repbase database with default parameters, and Blastn explores the NCBI database using default parameters. Deleted and truncated small copies can lead to false-positive results; we extracted copies only with query > 90% and similarity > 80%. We found very few good hits in Repbase as per the designed criteria for the study, but retrieved many homologous sequences from Blastn searched at NCBI. Many sequences belonging to the same lineages within the same species resulted in similar hits, so we selected sequences with the highest DNA similarity throughout the length as a representative sequence from each lineage, as explained above. The top-ranking results from NCBI Blastn, i.e., query coverage > 90%, identity > 85% with lowest E-value, and higher bit score, are shown in Table 1. Likewise, the top-ranking results from RepBAse, i.e., query coverage > 90%, similarity with consensus sequence > 90%, Pos-value, and higher bit score, are shown in Table 2. The detected sequences in other insects display higher nucleotide identity, which exceeds the expected identity values when comparing transposable elements in distantly related species. We found many sequences in the NCBI and RepBase databases to be very similar to *Rhus* gall aphids transposons (identity ≥ 85%, and query ≥ 95%) belonging to seventeen insects species from five different orders, i.e., (Hymenoptera, Diptera, Coleoptera, Lepidoptera, and Neuroptera). The *Rhus* gall aphid species belongs to the order Hemiptera of Class Insecta, so the current study represents HTT events among six different orders of Class Insecta (Table 1 and Table 2).

**Figure 2:**
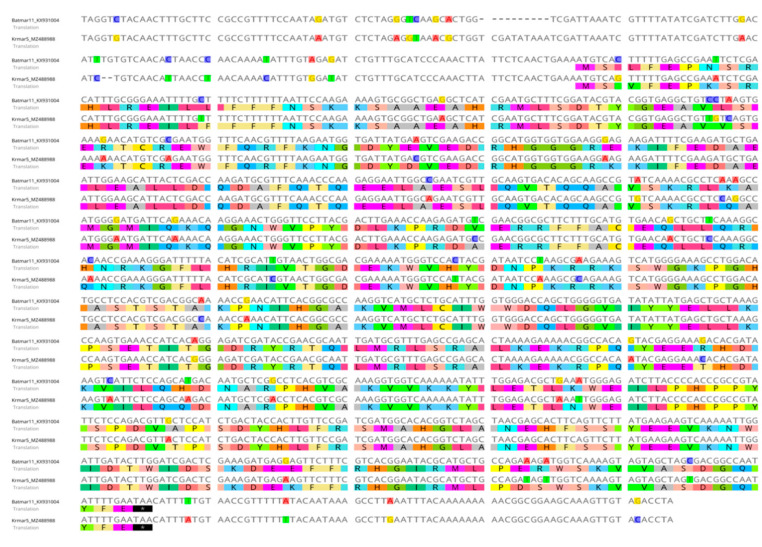
Pairwise alignment of nucleotide and amino acids sequences of *Rhus* gall MLE (Krmar5) from *Kaburagia rhusicola* and *Bactrocera tryoni* MLE (Batmar11) with intact ORF for transposase, showing high pairwise similarity (94.6%) throughout nucleotide length and pairwise similarity (96.1%) throughout amino acid sequence, and >96% similarity between the TIRs.

**Figure 3:**
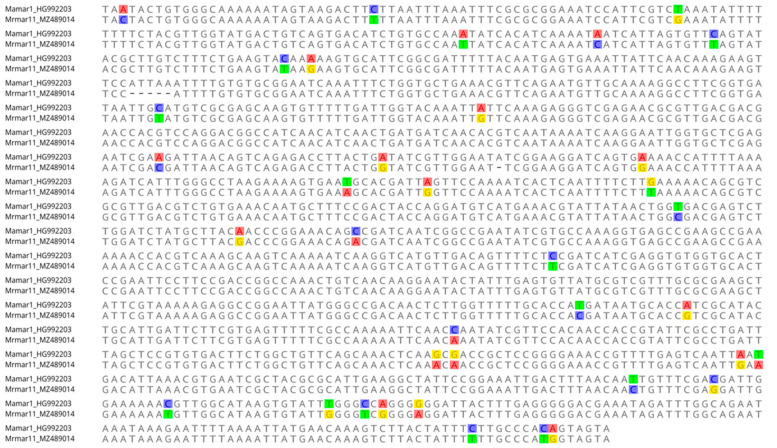
Pairwise alignment of nucleotide sequences of MLEs from *Rhus* gall aphid *Melpahis rhois* (Mrmar11) and butterfly *Mellicta athalia* (Mamar1), showing high similarity (96.80%) throughout the length of sequences, with no intact ORF, and more than > 93% similarity for TIRs.

As expected for TEs, in some cases, the extent of sequence identity was observed throughout the length of elements of TEs, including TIRs, which strongly supports the hypothesis of the HTT event. Some of the examples that support this can be seen in Figure 2 and Figure 3 and Table 1 and Table 2, in which the DNA sequence similarity between TEs of *Rhus* gall aphid species with distantly related species of different orders are more than 96%, while the amino acid sequences similarities are also more than 96%.

### 3.5. Phylogenetic Analysis of HTT among Rhus Gall Aphids and Other Insects

**Figure 4:**
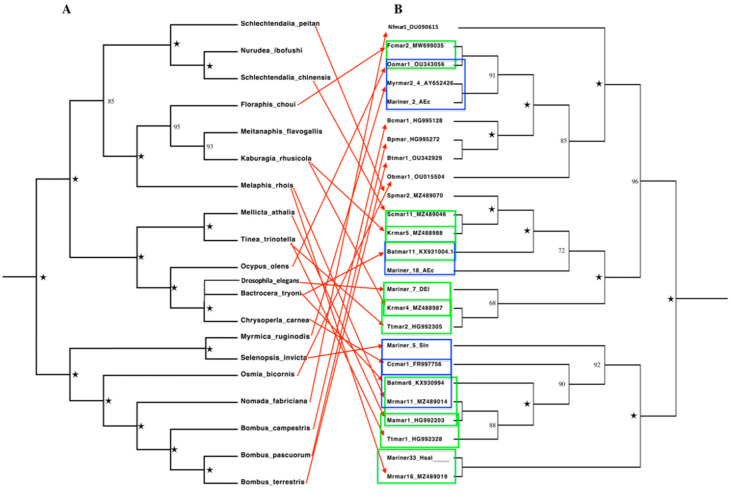
Comparison of species tree with MLEs tree to infer HTT events between *Rhus* gall aphids and other insects. (**A**) Left side species tree of seven *Rhus* gall aphids and thirteen insects belongs to five different orders, constructed based on 20 genes. (**B**) Phylogenetic tree of *Rhus* gall MLEs and extracted MLEs from other insects’ species. The green rectangles indicate HTT events between *Rhus* gall MLE and other insects of a different order. In contrast, the blue rectangle shows HTT events involving *Rhus* gall aphids and between insects of different orders in this study. Red arrows indicate the position of each MLE of each species and describe the highly patchy distribution of the MLEs. Five-pointed star represent 100% bootstrap value.

To further confirm the phenomenon of HTT between *Rhus* gall aphids and five distantly related insect orders, we constructed the species tree (See Figure 4A) of all included species in this study by selecting 20 highly conserved orthologous genes, resulting in a good quality phylogenetic tree. We could not retrieve the mitochondrial and nuclear genes for *Drosophila elegans* and *Herpegnathos saltator* due to unavailability in public databases. *Drosophila elegans* was branched manually in the species tree using its divergence information from Timetree online, while the position of *Herpegnathos saltator* can be represented by other ant species, i.e., *Acromyrmex echinatior*. We concatenate and aligned the selected 20 genes of the species; 15 mitochondrial genes, i.e., 12S rRNA, 16S rRNA, ATP6, APT8, COX1, COX2, COX3, Cyt-b, ND1, ND2, ND3, ND4, ND4L, ND5, and ND6, and 5 nuclear genes, i.e., Long-wavelength rhodopsin resistance gene (*lwrh*), wingless (*wnt-1*), Elongation factor 1-alpha (*EF1-alpha*), Histone (*H3*), and 18S rRNA gene (see Supplementary File S2) to construct species tree. We also constructed the transposons tree (Figure 4B) of representative sequences of *Rhus* gall aphids MLEs and other insects MLEs recovered from NCBI GenBank and Repbase.

We compared the MLEs tree to the species tree of the *Rhus* gall aphids and other insects included in this study (Figure 4). These comparisons clearly showed at least five events of HT among *Rhus* gall aphids and other insects belonging to five different orders and few events of HTT within the insects of the other five orders. Seven MLEs from three subfamilies and four lineages of *Rhus* gall aphids clustered with the MLEs of insects from distantly related orders provide strong evidence of HTT. For instance, an MLE (Fcmar2_MW699035) of the gall aphid *Floraphis choui* belonging to the *Botmar*-like lineage of *Mauritiana* subfamily clustered with these of *Ocypus olens* (Oomar1_OU343056) from Coleoptera order, *Myrmica ruginodis* (Myrmar2_AY652426), and other insects from *Bombus* genus of order Hymenoptera. In the present scenario, MLEs of *Rhus* gall aphid nested with the MLEs of very distantly related orders, i.e., Coleoptera and Hymenoptera, diverged between 323–392 MYA from order Hemiptera, which provides strong evidence of HT between species of these orders. Similarly, MLEs of *Kaburagia rhusicola* (Krmar4 & Krmar5) nested with MLEs of *Drosophila elegans* (*Mariner*_7_DEI), *Bactrocera tryoni* (Batmar11_KX931004.1) from order Diptera, and *Tinea trinotella* (Ttmar2_HG992305) from order Lepidoptera, respectively. Other MLEs from *Rhus* gall aphid *Melaphis rhois* (Mrmar11) clustered with these of *Chrysoperla carnea* (Ccmar1_FR997756) from order Neuroptera, *Bactrocera tryoni* (Batmar6_KX930994) from order Diptera, and *Mellicta athalia* (Mamar1_HG992328) from order Lepidoptera. At the same time, one MLE (Mrmar16) nested with that of *Herpegnathos saltator* (Mariner33_Hsal) from the Hymenoptera order (Figure 4B).

**Figure 5:**
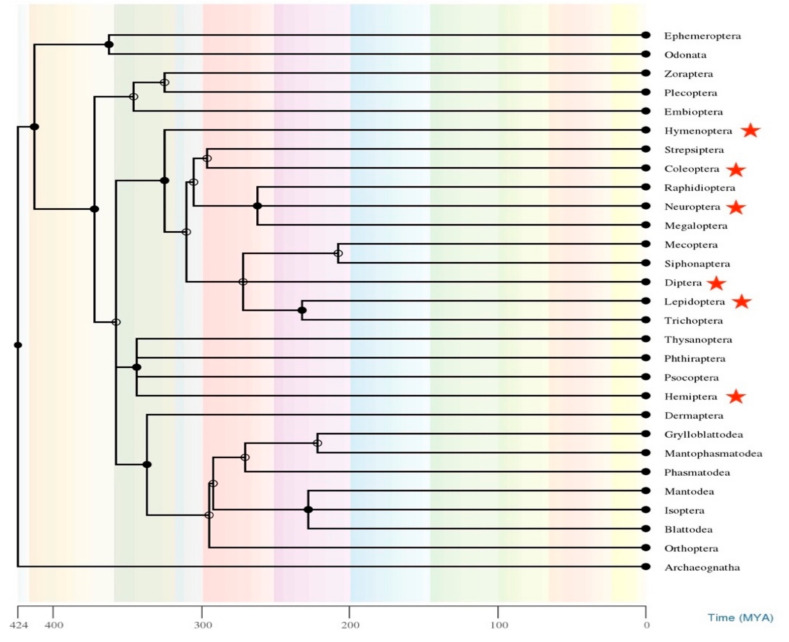
Phylogenetic tree constructed with time tree online, showing the relation and divergence time (MYA) between the insects’ orders. Five-pointed stars at the tip of the branch indicate the orders included in the study, which are involved in HTT.

All the species mentioned above diverged from *Rhus* gall aphids with more than 300 MYA (Figure 5). The genes and MLEs of these species cannot nest together in a phylogenetic tree in normal circumstances in the absence of HT. In conclusion, of the above phylogenetic analysis, the clustering of MLEs from five distantly related orders of insects with the *Rhus* gall aphids MLEs provides strong evidence of HT between these groups of insects.

Several HTTs events have also been observed within the other insect orders included in this study. For instance, a *Botmar*-like MLE from the *Mauritiana* subfamily (Oomar1_OU343056) extracted from *Ocypus olens* of Coleoptera order nested with the MLEs of *Myrmica ruginodis* and *Acromyrmex echinatior* of order Hymenoptera, which reflects multiple events of HTT of this MLE between *Myrmica ruginodis*, *Ocypus olens*, and *Floraphis choui* (Figure 4B). Another such event of HTT can be observed in a *Batmar-like* MLE of *Mauritiana* subfamily in *Acromymex echinatior* (*Mariner*-18_Ace) of Hymenoptera order, which nested with *Bactrocera tryoni* MLE (Batmar-11_KX931004) of order Diptera. It also reflects multiple events of HTTs of these elements between *Acromymex echinatior*, *Bactrocera tryoni*, and *Kaburagia rhusicola*. Furthermore, one MLE of the *Drosophila* subfamily (Ccmar_FR997756) extracted from *Chrysoperla carnea* (Order: Neuroptera) was nested with Batmar6_KX930994 of *Bactrocera tryoni* (Order: Diptera), Mamar1_HG992203 of *Mellicta athalia*, and Ttmar1_HG992328 of *Tinea trinotella* from order Lepidoptera, which indicates several events of HTT by this MLE between *Chrysoperla carnea*, *Bactrocera tryoni*, *Mellicta athalia*, *Tinea trinotella*, and *Melaphis rhois* (Figure 4B).

We could not discriminate the HTT events within *Rhus* gall aphid species. All the aphids in this study are closely related and belong to the same subfamily Eriostominae, with closed divergence time. As recently diverged species share high nucleotide similarities in their sequences, making it challenging to detect HT in them based on nucleotide identities. Although the *Rhus* gall MLEs showed patchy distribution among the *Rhus* gall aphids, but did not produce positive HT signals based on the designed criteria for the present study.

In conclusion, the above results show that *Rhus* gall aphid transposons had striking identities with transposons of distantly related species and clustered together in the MLEs tree. Meanwhile, all the *Rhus* gall aphids clustered apart from other insects in the species tree based on nuclear and mitochondrial genes. These unexpected sequence similarities of galling aphids MLEs with other insects from different orders and the uneven distribution of MLEs in the phylogenetic tree are assumed to be solid evidence of HTT between *Rhus* gall aphids and other insects.

### 3.6. Estimation of Divergence Time

All of the insects in the tree shared a common ancestor and belonged to the same Class; we estimated the divergence time of each species and the order of insects included in this study (Figure 5). Divergence times were estimated to discover the distances between the species, which will help to discriminate the HT events between the sequences. Due to evolution, species with common ancestors and that diverged a long time ago tend to accumulate more mutations and changes in their nucleotide sequences. We estimated the divergence time of all of the species using Timetree online at (https://www.timetree.org, accessed on 1 January 2022) and the divergence time between *Rhus* gall aphids with all of the other insects in the tree was more than 300–350 Myr, with an average of 325 Myr. Comparatively, the MLEs sequences of *Rhus* gall aphids and other insects showed contrasting nucleotide similarities, which was impossible for the neutrally evolving genes diverged so long ago. For example, *Sclechtendalia chinensis* diverged 350–375 Myr from *Bactrocera tryoni* and nested very far apart in the species tree. Still, their MLEs showed 96% nucleotide sequence similarities between them and nested as sister sequences in the MLEs tree. The very high divergence time between the studied species and closed MLEs sequence similarities further support our claim of HTT events between these species.

## 4. Discussion

HTT is a well-known and reported phenomenon documented in many metazoans, including insects. Although the exact mechanism of HTT is poorly understood, the geographic proximity and host-parasite interactions might help in the exchange of genetic material in distantly related species [49,50]. To date (visited 1 January 2022), there are 5689 cases of HTT that have been reported in the Horizontal transfer of transposons database (HTT-DB) [51]. Among all of the HTT events reported in HTT-DB, the *Tc1/Mariner* Superfamily of DNA transposons contribute to most cases, i.e., 2523 out of 4271 DNA transposon HT events. HTT is well reported in most insects. Recent studies uncovered thousands of HTT events in the Class Insecta [19], while others reported thousands of HTT events in vertebrates [52]. However, there is no report of HTT involving *Rhus* gall aphids to date due to the unavailability of TEs data in these aphids. We recently reported *Mariner* transposons in *Rhus* gall aphids for the first time. We followed the previous findings to uncover the events of HTT among *Rhus* gall aphids and other insects and closed the knowledge gap about HTT in galling aphids.

Methodologically, it is not easy to infer HTT events. Several tools and methods can be used; for example, the VHICA tool concludes HTT events based on codon usage analyses and compares synonymous and non-synonymous substitutions rates [18]. Unfortunately, there are limitations to these automatic tools. These perform poorly if the divergence between the species in which HTT is inferred increases, leading to substitution saturation, which causes loss of the phylogenetic signal [53]. In this study, the divergence time between *Rhus* gall aphids and other species for which HTT was inferred is huge (>300 Myr), and the methods based on phylogeny and genetic distance seem to be more suitable, which is also suggested by previous studies [54,55]. We followed a two-step approach explained above and uncovered a few events of HTTs between *Rhus* gall aphids and 11 other species of insects belonging to 5 orders of Class Insecta. HTT events detected from more than one species of the same order for the same MLEs were considered single HTT events. Among the seven species of *Rhus* gall aphids, TEs from three species seem to have undergone HTT events.

We have inferred HTT events involving six DNA transposons of the *Mariner* family in *Rhus* gall aphids. Our findings suggest that HTT events between *Rhus* gall aphids and distantly related insects might have occurred several times. Our results also suggest that the MLEs involved in *Rhus* gall aphids’ HTT events have also undergone many HT between other orders of insects. Moreover, the common ancestor of all the insects, *Rhus* gall aphids (Order: Hemiptera), is quite distantly related to other insects in this study, and the HTT scenario is very clear from the results; however, it is challenging to infer both the direction and the vector of the HTT events described in this study.

Interestingly, all of the putative cases of HTT detected in this study involved 21 insect species from five different orders. In this respect, Order Hymenoptera seems to be the preferred order in exchanging MLEs since seven other bee species belonging to three different genera and five ants species from different genera are putatively involved in HTT events with four *Rhus* gall aphids *Mariner* elements (namely Fcmar2, Krmar4, Krmar5, Mrmar16). Insects from the order Hymenoptera have been involved in many HTT events in previous studies [38], as is supported by the present study. In comparison, four of the twenty-one insects involved in HTT events in the study belong to the order Lepidoptera and four from the order Diptera, respectively, with five *Rhus* gall elements (Krmar4, Krmar5, Spmar2, Scmar11, Mrmar11). A recent study found that the Lepidoptera order has been the hotspot of HTT in insects [20], while fruitflies from the order Diptera were also suggested as good horizontal transfer candidates [56]. At the same time, one species, each from beetles (Order: Coleoptera) and Laecwings (order: Neuroptera), also seemed to be involved in the HTT event with *Rhus* gall aphid *Mariner* elements (Fcmar2, Mrmar11). All the *Mariner* elements involved in HTT events in this study belong to two subfamilies in which elements of the *Mauritiana* subfamily seem to be dominantly involved in HTT events.

Although there is a patchy distribution of MLEs among the *Rhus* gall aphids, and they do not follow the species tree, it is difficult to infer the HTT events among closely related species [19]. All the *Rhus* gall aphids belong to the same subfamily Eriosomatinae and are closely related phylogenetically. To avoid false-positive results, we could not infer HTT events within *Rhus* gall aphids but have drawn observations from the study that *Rhus* gall genomes are equally targeted to transposition and HT events of transposons. The presence of potentially active TEs in the genome could also be involved in HT events within *Rhus* gall aphids yet be undetectable due to a closed evolutionary relationship.

## 5. Conclusions

Our study reveals that the evolutionary history of *Mariner* transposons in *Rhus* gall aphids has been subjected to many events of HT, involving a total of five other orders of insects at the same time. Moreover, our results show that *Mariner* elements from the *Mauritiana* subfamily are involved in more HT events compared to other MLEs. These results contribute to the description of transposons as genomic symbionts that mobilize and move between different host lineages, evolving and shaping their host genomes. Our study represented the HT events involving the *Rhus* gall aphids for the first time and closed the information gap about HTs events in galling aphids.

## Figures and Tables

**Table 1:** The top-ranking results from NCBI Blastn, i.e., query coverage > 90%, identity > 85% with lowest E-value, and higher bit score along with target species and their respective order.

*Rhus* Gall Aphid Species	*Rhus* Gall Aphids MLE	Target Species Element	Species Order	Target Accession	NCBI Blastn Alignment Parameters			
					% Query Coverage	%Identity	E-Value	Bit Score
*Floraphis choui*	Fcmar2	*Myrmica ruginodis*	Hymenoptera	AY652426	99	90.10	0	1657
		*Bombus campestris*	Hymenoptera	HG995146	100	87.66	0	1489
		*Nomada fabriciana*	Hymenoptera	OU015690	99	87.48	0	1476
		*Ocypus olens*	Coleoptera	OU343056	99	87.55	0	1469
		*Bombus terrestris*	Hymenoptera	OU342929	99	87.39	0	1469
		*Bombus pascuorum*	Hymenoptera	HG995272	99	87.25	0	1454
		*Osmia bicornis*	Hymenoptera	OU015504	99	85.60	0	1339
*Kaburagia rhusicola*	Krmar4	*Tinea trinotella*	Lepidoptera	HG992316	99	85.39	0	1315
	Krmar5	*Bactrocera tryoni*	Diptera	KX931004	100	94.6	0	1750
*Meitanaphis flavogallis*	Mfmar7	*Bactrocera tryoni*	Diptera	KX931004	100	93.57	0	1720
*Melpahis rhois*	Mrmar11	*Mellicta athalia*	Lepidoptera	HG992203	99	96.80	0	2185
		*Bactrocera tryoni*	Diptera	KX930994	100	95.67	0	2108
		*Chrysoperla carnea*	Neuroptera	FR997756	99	91.56	0	1783
		*Tinea trinotella*	Lepidoptera	HG992328	99	90.98	0	1307
*Schlechtendalia chinensis*	Scmar11	*Bactrocera tryoni*	Diptera	KX931004	100	93.64	0	1910
*Schlechtendalia peitan*	Spmar2	*Bactrocera tryoni*	Diptera	KX931004	100	94.49	0	1910

**Table 2:** The top-ranking results from RepBase, i.e., query coverage >90%, identity >85% with consensus sequence, and higher bit score along with target species and their respective order.

*Rhus* Gall MLEs	*Rhus* Gall Aphids	Target Species Name	Order	Target MLE Name	Query	Similarity to (Consensus)	Bit Score
Mrmar16	*Melaphis rhois*	*Herpegnathos saltator*	Hymenoptera	*Mariner*-33_HS al	92%	0.8307 (>97%)	7128
Fcmar2	*Floraphis choui*	*Acromyrmex echinatior*	Hymenoptera	*Mariner*-2_AEc	99%	0.8925 (89.2%)	8806
Krmar4	*Kaburagia rhusicola*	*Drosophila elegans*	Diptera	*Mariner*-7_DEL	95%	0.8003 (~96%)	6914
Krmar5	*Kaburagia rhusicola*	*Acromyrmex echinator*	Hymenoptera	*Mariner*-18_AEc	100%	0.8213 (~96%)	7732
Mrmar11	*Melaphis rhois*	*Solenopsis invicta*	Hymenoptera	*Mariner*-5_ Sin	100%	0.8088 (>97%)	6926

## Data Availability

All the data used in this study are present in the manuscript and Supplementary Materials.

## Supplementary Materials

The following supporting information can be downloaded at: https://www.mdpi.com/article/10.3390/biology11050731/s1, Supplementary File S1: Excel spreadsheet containing all the detailed information of the *Rhus* gall aphids MLEs, Supplementary File S2: CSV spreadsheet containing nucleotide sequences of the 20 genes of all insects used for the construction of species tree, Supplementary File S3: CSV file contains the *Mariner* elements extracted from the insect’s species and used in the construction of the tree, and a table showing the genetic distances between them, Supplementary File S4: CSV spreadsheet containing the alignments used to construct all the phylogenetic trees in this study.
